# Nested likelihood-ratio testing of the nonsynonymous:synonymous ratio suggests greater adaptation in the piRNA machinery of *Drosophila melanogaster* compared with *Drosophila ananassae* and *Drosophila willistoni*, two species with higher repeat content

**DOI:** 10.1093/g3journal/jkaf017

**Published:** 2025-02-21

**Authors:** Justin P Blumenstiel, Sarah B Kingan, Daniel Garrigan, Tom Hill, Jeffrey Vedanayagam

**Affiliations:** Department of Ecology and Evolutionary Biology, University of Kansas, 1200 Sunnyside Avenue, Lawrence, KS 66045, USA; Pacific Biosciences, Long Read DNA Applications, 1305 O’Brien Drive, Menlo Park, CA 94025, USA; Wisdom Panel, 18101 SE 6th Way, Vancouver, WA 98683, USA; Department of Ecology and Evolutionary Biology, University of Kansas, 1200 Sunnyside Avenue, Lawrence, KS 66045, USA; Axle Informatics, 6116 Executive Blvd, Suite 400, Bethesda, MD 20852, USA; Department of Neuroscience, Developmental and Regenerative Biology, University of Texas at San Antonio, San Antonio, TX 78249, USA

**Keywords:** transposable elements, piRNA, *Drosophila melanogaster*, *Drosophila willistoni*, *Drosophila ananassae*, evolutionary arms race, evolutionary trench warfare, genetic conflict, FlyBase

## Abstract

Numerous studies have revealed a signature of strong adaptive evolution in the piwi-interacting RNA (piRNA) machinery of *Drosophila melanogaster*, but the cause of this pattern is not understood. Several hypotheses have been proposed. One hypothesis is that transposable element (TE) families and the piRNA machinery are co-evolving under an evolutionary arms race, perhaps due to antagonism by TEs against the piRNA machinery. A related, though not co-evolutionary, hypothesis is that recurrent TE invasion drives the piRNA machinery to adapt to novel TE strategies. A third hypothesis is that ongoing fluctuation in TE abundance leads to adaptation in the piRNA machinery that must constantly adjust between sensitivity for detecting new elements and specificity to avoid the cost of off-target gene silencing. Rapid evolution of the piRNA machinery may also be driven independently of TEs, and instead from other functions such as the role of piRNAs in suppressing sex-chromosome meiotic drive. We sought to evaluate the impact of TE abundance on adaptive evolution of the piRNA machinery in *D. melanogaster* and 2 species with higher repeat content—*Drosophila ananassae* and *Drosophila willistoni*. This comparison was achieved by employing a likelihood-based hypothesis testing framework based on the McDonald–Kreitman test. We show that we can reject a faster rate of adaptive evolution in the piRNA machinery of these 2 species. We propose that the high rate of adaptation in *D. melanogaster* is either driven by a recent influx of TEs that have occurred during range expansion or selection on other functions of the piRNA machinery.

## Introduction

Transposable elements (TE) are genetic parasites that proliferate in genomes despite causing damage (Hickey 1982). Because of this damage, mechanisms of TE suppression have evolved. A key mechanism of TE suppression is based on small RNAs known as piwi-interacting RNAs (piRNAs) (reviewed in Czech and Hannon 2016; Ozata *et al*. 2019; Kelleher *et al*. 2020; Onishi *et al*. 2021; Loubalova *et al*. 2023; Wang *et al*. 2023). For reasons that are poorly understood, there is a strong signature of rapid adaptation in proteins of the piRNA machinery of *Drosophila* (Vermaak *et al*. 2005; Heger and Ponting 2007; Obbard, Gordon, *et al*. 2009; Obbard, Welch, *et al*. 2009; Kolaczkowski *et al*. 2011; Lee and Langley 2012; Simkin *et al*. 2013; Lewis *et al*. 2016; Palmer *et al*. 2018). Several hypotheses have been proposed to explain this observation. One hypothesis is that the piRNA machinery is involved in a co-evolutionary arms race with TEs, perhaps due to TEs that antagonize mechanisms of genome defense (Vermaak *et al*. 2005; Obbard, Gordon, *et al*. 2009; Lee and Langley 2012; Parhad *et al*. 2017, 2020; Parhad and Theurkauf 2019). A related hypothesis, not invoking co-evolution, is that the piRNA machinery constantly adapts to strategies employed by new TEs (Clark and Kidwell 1997; Peccoud *et al*. 2017; Schwarz *et al*. 2021; Senti *et al*. 2023). The genomic autoimmunity hypothesis is that adaptation is driven by a fluctuation in the genomic TE burden (Blumenstiel *et al*. 2016) and the cost of off-target gene silencing (Olovnikov *et al*. 2013; Shpiz *et al*. 2014; Lee and Karpen 2017; Choi and Lee 2020; Miller *et al*. 2023). Higher rates of adaptation in the piRNA pathway may also be independent of TEs. For example, adaptation may be driven by the role of the piRNA machinery in suppressing meiotic drive. For instance, core mutations in the core piRNA pathway factor *aub* enhance meiotic drive mediated by the *Segregation Distortion* system in *Drosophila melanogaster* (Gell and Reenan 2013). Y-linked piRNAs also silence *Stellate* genes on the X-chromosome, that appear to be “ancient” meiotic drivers (Aravin *et al*. 2001, 2004; Courret *et al*. 2019; Chen *et al*. 2025).

Under a simple co-evolutionary arms race, one might predict that proteins of the piRNA machinery would adapt at a higher rate in species with greater TE content. Under the genomic autoimmunity hypothesis, one might instead predict similar rates in species with high and low TE content since what matters is the fluctuation rather than the current TE burden. Interestingly, a previous study (Castillo *et al*. 2011) indicated that the rate of evolution in the piRNA machinery (in *dN/dS*) is *slower* in species with higher TE content. However, a limitation of that study was that estimates for *dN/dS* were obtained from divergence times corresponding to tens of millions of years, but genomic TE estimates corresponding to current time. A better approach to measuring recent selection on the piRNA machinery is to use a McDonald–Kreitman (MK) test (McDonald and Kreitman 1991). Using this approach, we sought to compare evolution of the piRNA pathway in *D. melanogaster* to that of species with much greater TE content: *Drosophila ananassae* and *Drosophila willistoni.*

Several challenges arise from comparing patterns of adaptation across species using the MK test. Demography and divergence times can have a complex impact on patterns of polymorphism and divergence (Ohta 1993; Welch 2006; Hughes 2007; Elyashiv *et al*. 2010; Akashi *et al*. 2012; Keightley and Eyre-Walker 2012). Also, comparing the distributions of summary statistics based on ratios can be challenging when there are low values in denominators (Stoletzki and Eyre-Walker 2011). Hence, we employed a simplified nested likelihood-framework to compare patterns of divergence and polymorphism across species. Rather than estimate selection coefficients or compute summary statistics, we developed an approach to ask a simpler question: For species with higher TE content, is there a significantly greater excess of nonsynonymous divergence in the piRNA machinery (relative to polymorphism) compared with all other genes? This approach enables a hypothesis testing framework based on the likelihood-ratio test (LRT) using the raw data of divergence and polymorphism.

## Materials and methods

### Strains and sequencing

For *D. melanogaster* population genomic data, we obtained next-generation sequencing data from NCBI (Pool *et al.* 2012; Supplementary Table S1). The *Drosophila simulans* near-outgroup used in analyses is from the w501 strain (Hu *et al*. 2013). The *Drosophila yakuba* far-outgroup used in our analyses is the publicly available genome assembly available on Flybase (Clark *et al*. 2007). *D. ananassae*, *Drosophila bipectinata*, and *Drosophila atripex* strains were kindly provided by the UCSD stock center and Artyom Kopp. *D. willistoni*, *Drosophila paulistorum*, and *Drosophila nebulosa* strains were kindly provided by the UCSD stock center, Jenny Gleason and Jeff Powell. Additional strain information is provided in Supplementary Table S1. DNA was extracted with the 96-well Qiagen DNeasy Blood and Tissue Kit. Libraries were prepared with the Illumina Nextera kit and 100 bp paired end sequencing was performed on an Illumina HiSeq2000.

### Read mapping, alignments, and sequence analysis

Reference-based genome assemblies of 6 European and 9 sub-Saharan African strains of *D. melanogaster* were generated using next-generation short-read data from the NCBI SRA (Pool *et al.* 2012). A list of strains and their respective SRA IDs are provided in the Supplementary Table S1. We downloaded the SRA files using the fastq-dump tool and independently evaluated and verified the read qualities using FastQC. Paired-end reads were then mapped to the *D. melanogaster* genome assembly (version r6.28) using the “aln/sampe” functions of the BWA aligner using default settings (Li and Durbin 2009). Resulting individual BAM files were merged and sorted with SAMTOOLS (Li *et al.* 2009). Variants were called using the POPBAM software with default settings (Garrigan 2013). Gene alignments were then constructed for the longest transcript per gene from the FlyBase mRNA annotations, using the PERL script, PBsnp2fa.pl (https://github.com/skingan/PBsnp2fa.pl). For *D. melanogaster*, ancestral and derived states were then inferred by constructing a gene-by-gene alignment for every gene using *D. simulans* w501 strain (Hu *et al*. 2013) as a near-outgroup and *D. yakuba* strain Tai18E2 (Clark *et al*. 2007) as a far-outgroup. The CDS for every gene was extracted from the population genomic data after the polymorphisms were reconstituted from the POPBAM output. Individual genes were then extracted into FASTA format to provide alignment-ready FASTA entries for every gene, along with a near and far outgroup orthologs. The alignment was performed using MUSCLE aligner (Edgar 2004), and the alignments were created with respect to the *D. melanogaster* reference as a template. Thus, if there were any gaps in the *D. simulans* or *D. yakuba* outgroup, those sequences were removed in all species, ensuring that the alignment is in-frame after removing gaps. Likewise, if there were gaps in *D. melanogaster* reference due to insertions in *D. simulans* or *D. yakuba*, those nucleotides were removed in all species to maintain an in-frame alignment. The gaps were removed using the “remove_columns” (gaps) function implemented in the Bio::AlignIO CPAN module. The resulting alignments were thoroughly inspected for all piRNA pathway genes, and the other alignments were manually spot-checked for quality using the Geneious software. These gene-by-gene alignments were then used to obtain variant counts using the CDS2SFS.pl PERL script (https://github.com/skingan/CDS2SFS), providing an unfolded site-frequency spectrum (SFS).

For *D. ananassae* and *D. willistoni*, we collected population genomic data by performing Nextera 100 bp paired-end Illumina sequencing. We constructed reference-based genome assemblies by aligning the paired-end reads to reference genomes for *D. ananassae* (version r1.05) and *D. willistoni* (version r1.05) downloaded from FlyBase (Öztürk-Çolak *et al.* 2024), using methods described above for our *D. melanogaster* analyses. For *D. ananassae* gene-by-gene alignments, *D. atripex* was used as a near outgroup and *D. bipectinata* was used as a far outgroup. Similarly, for *D. willistoni*, *D. paulistorum* was used as a near outgroup and *D. nebulosa* was used as a far outgroup. Finally, we obtained variant counts and SFS using the CDS2SFS.pl PERL script.

## Statistical analysis

### Likelihood model

Analysis was restricted to strict 1:1:1 orthologs. Unfolded SFS values were collapsed into 2 classes—nonsynonymous and synonymous. The grand sum of nonsynonymous and synonymous variants (both polymorphic and divergent and across all 3 species) was used to calculate the baseline global nonsynonymous:synonymous (N:S) odds. Singletons were retained since it is not clear how a uniform cutoff could be employed across species with different population histories and divergence times. Moreover, while low-frequency mildly deleterious variants can lead to biased estimates of the fraction of nonsynonymous substitutions that are adaptive, the objective of this work is hypothesis testing rather than parameter estimation. From a set of 8,747 1:1:1 orthologs, 31 genes from (Ozata *et al*. 2019) were selected to represent the piRNA pathway. The cytoplasmic components were *aub*, *fs(1)Yb*, *shu*, *spn-E*, *vls*, *csul*, *Hen1*, *tej*, *krimp*, *BoYb*, *armi*, *SoYb*, *ago3*, *zuc*, *vret*, *qin*, *minotaur*, *papi*, *Gasz,* and *Nbr.* The nuclear components were *piwi*, *mael*, *del*, *cuff*, *rhino*, *wde*, *Panx*, *arx*, *moonshiner*, *bootlegger,* and *Nxf2*. *Vasa*, *tapas*, *tudor*, *UAP56*, *Hsp83,* and *Ci* were excluded either due to challenges in detecting clear orthologs or concerns about overlapping non-piRNA function.

The likelihood model was implemented in R (Supplementary Material S2). Using a likelihood framework allows a flexible approach to nested hypothesis testing because any number of nested models can be compared. Maximum-likelihood (ML) NS:S odds estimates were obtained by using the probability of success *P*, equal to odds/(1 + odds) and the likelihood of the data for a given gene was determined using the binomial probability. For example, for a gene with 4 nonsynonymous (NS) polymorphisms and 2 synonymous (S) polymorphisms, conditional on 6 (N) variants, the ML estimate of the NS:S odds are 4:2 (2) and the ML estimate of *P* = 2/3. In turn, the likelihood under the ML estimate is obtained using the binomial probability, as such:


$$\frac{N!}{\mathrm{NS}!(N-\mathrm{NS})!}p^{\mathrm{NS}}(1-p{)}^{\left(N-\mathrm{NS}\right)}=\;\frac{6!}{4!2!}2/3^{4}1/3^{2}$$

For the entire data set, a global NS:S odds estimate *α* (0.308) was obtained by summing all nonsynonymous and synonymous variants, both for divergence and polymorphism, across all 3 species. This global estimate is not to be interpreted as an average value across all individual genes since some genes will have many more counts than others and contribute more to this estimate. Rather, it is to be considered a baseline that an odds modifier *β* adjusts accordingly for different partitions of the data. For a single gene, as above, the likelihood, with *P* being β*α/(β*α+1) , is given as:


$$L(\beta |\alpha ,\mathrm{NS},N)=\frac{N!}{\mathrm{NS}!(N-\mathrm{NS})!}{\left(\frac{\beta \;*\;\alpha }{\beta \;*\;\alpha +1}\right)}^{\mathrm{NS}}{\left(1-\left(\frac{\beta \;*\;\alpha }{\beta \;*\;\alpha +1}\right)\right)}^{\left(N-\mathrm{NS}\right)}$$

where NS is the nonsynonymous variant count and (N – NS) is the synonymous variant count for either divergence or polymorphism. The ML estimate of modifier *β* (and model likelihood) was determined through numerical optimization of the log-likelihood function, summed in a gene-wise manner, in R using the optim() function. For genes where both nonsynonymous and synonymous values were 0, the likelihood (conditional on *N* = 0) was set equal to 1 (log-likelihood = 0) and the summed log likelihood is given by iterating over all genes, across divergence, polymorphism, species and class (piRNA or not piRNA). For a single value of *β* across the entire data set, this would be given as:


$$\ln(L(\beta |\alpha ,\mathrm{NS},N))=\underset{i}{\sum }\;\underset{j}{\sum }\;\underset{k}{\sum }\;\underset{l}{\sum }\;\ln(L(\beta |\alpha ,NS_{ijkl},N_{ijkl}))$$

where the summation of *i*, *j*, *k,* and *l* is over all 3 species (*i*), with respect to divergence and polymorphism (*j*), with each class (*k*, piRNA or non-piRNA) across all genes within the respective gene class (*l*).

Values of *β* could then be evaluated for different partitions of the data. For example, to consider a specific single value of *β* for each species, ignoring the difference between polymorphism and divergence (*j*) and gene class (*k*), where *l* iterates over all genes, the full log-likelihood would be equal to:


$$\ln(L(\beta_{\mathrm{mel}},\beta_{\mathrm{ana}},\beta_{\mathrm{wil}}|\alpha ,\mathrm{NS},N))=\underset{j}{\sum }\;\underset{k}{\sum }\;\underset{l}{\sum }\;\ln(L(\beta_{\mathrm{mel}}|\alpha ,NS_{\mathrm{mel},jkl},N_{\mathrm{mel},jkl}))+\underset{j}{\sum }\;\underset{k}{\sum }\;\underset{l}{\sum }\;\ln(L(\beta_{\mathrm{ana}}|\alpha ,NS_{\mathrm{ana},jkl},N_{\mathrm{ana},jkl}))+\;\underset{j}{\sum }\;\underset{k}{\sum }\;\underset{l}{\sum }\;\ln(L(\beta_{\mathrm{wil}}|\alpha ,NS_{\mathrm{wil},jkl},N_{\mathrm{wil},jkl}))$$

As expected, for the entire data set, the single ML estimate for *β* was essentially 1 (Fig. 2a). Estimates of *β* were further obtained for different partitions of the data indicated by subscript. For example, *β*_ana.wil.div_ would indicate the *NS:S* modifier value for divergence for *D. ananassae* and *D. willistoni* alone, for all genes irrespective of class (piRNA or non-piRNA).

Model testing was performed by comparing the likelihoods under different models, with the likelihood test statistic equal to 2(ln(Max.LikeModel2)−ln(Max.LikeModel1) and the *P*-value determined using the *χ*^2^ distribution with degrees of freedom equal to the number of additional parameters.

### Hypothesis testing by bootstrap

Bootstrap support was obtained with gene-wise sampling with replacement for the entire data set, permuting the counts for the 31 piRNA genes and the rest of the genes separately. For each of these permutations, ML estimates of *χ* and *ζ* were obtained. A *P*-value for a difference was determined considering the proportion of gene-wise permutations that showed a contrast in the opposing direction of the ML estimates from the unpermuted data.

## Results

Compared with *D. melanogaster*, *D. ananassae*, and *D. willistoni* have greater TE abundance (Clark *et al*. 2007; Kim *et al*. 2021). Therefore, we performed branch-specific MK-like analysis from these 3 species to compare patterns of recent divergence in the piRNA machinery. For *D. melanogaster*, we used available sequences (Pool *et al.* 2012; Supplementary Table S1). For *D. ananassae* and *D. willistoni*, we sequenced strains kindly provided by Artyom Kopp and Jeff Powell (Strain information in Supplementary Table S1). For *D. melanogaster*, branch-specific substitutions were estimated using *D. simulans* as a near outgroup and *D. yakuba* as a far outgroup. For *D. ananassae*, *D. atripex* was used as a near outgroup and *D. bipectinata* was used as a far outgroup. For *D. willistoni*, *D. paulistorum* was used as a near outgroup and *D. nebulosa* was used as a far outgroup. From these sequences, nonsynonymous and synonymous polymorphism and branch-specific divergence was measured for each species for 8,747 strict 1:1:1 orthologs, including 31 genes that are components of the piRNA machinery (Supplementary Table S3).

**Fig. 1.**
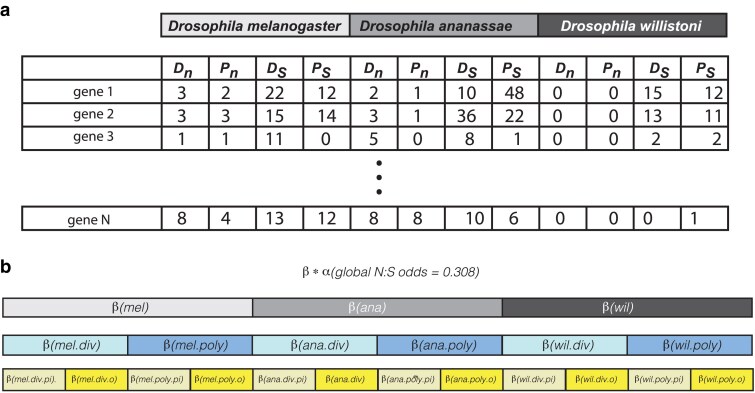
Data structure and model testing approach. a) Structure of data. For each gene (piRNA and other), counts of nonsynonymous divergence (Dn), nonsynonymous polymorphism (Pn), synonymous divergence (Ds) and synonymous polymorphism (Ps) were determined. b) Structure of nested model testing. A baseline N:S odds (*α*) from the entire data set was established and nested modifiers (*β*) were evaluated using the binomial. div, divergence; poly, polymorphism; pi, piRNA genes; o, other genes.

Hypothesis testing was performed using a likelihood-ratio based framework (Fig. 1) by determining the likelihood of the data (Fig. 1a) under different estimates of the N:S odds. Likelihoods were calculated using the binomial, with the estimated N:S odds value being used to determine binomial


Probabilities(P=odds/(1+odds))

The simplest version of the model estimated a single odds parameter for all genes and species, equal to the cumulative sum (both polymorphic and divergent) for nonsynonymous variants divided by the cumulative sum of synonymous variants. This global odds estimate (nonsynonymous: synonymous) was equal to 0.308 and was used as a baseline that was scaled considering additional factors. We first tested whether this global N:S odds varied across species. This involved calculating the likelihood of the data under models in which each species had a single modifier of the baseline odds estimate. The likelihood of the data (Fig. 1a) was maximized by adjusting the species level odds modifier (Fig. 1b, in gray). This approach was further extended to consider additional modifier parameters. The most parameter rich model was that which included the baseline odds estimate and nested modifier parameters down to the level of species (3 parameters), divergence/polymorphism (2 parameters), and piRNA/other (2 parameters, for a total of 12 additional parameters).

**Fig. 2.**
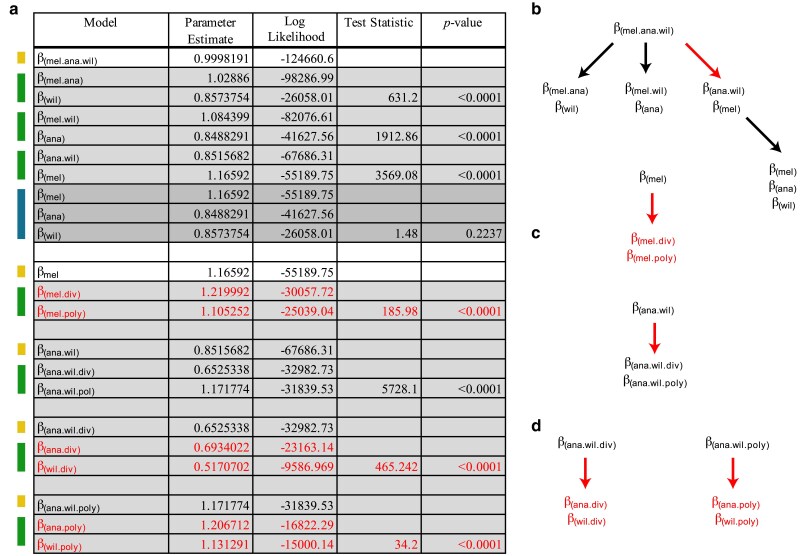
Model testing for differences in the N:S odds across each of the 3 species with respect to divergence and polymorphism. a) Colors on the left indicate nested models being tested against models indicated above. Yellow indicates a single parameter model, green indicates a 2-parameter model and blue indicates a 3-parameter model. Three-parameter models were tested against the 2-parameter model with the highest likelihood. *P*-values were determined based on the LRT statistic, using the *χ*^2^ distribution with degrees of freedom equal to the number of additional parameters. In all cases, the number of additional parameters for each test was equal to 1. Red indicates the parameters of the final model selected. Here, a distinct value for N:S odds was determined for divergence and polymorphism for each species (6 parameters). b–d) Structure of LRT. Red arrows indicate a nested LRT that favors an additional parameter. Red text indicates the final selected parameters for the chosen model. b) Considering a single N:S odds across the whole data set, testing supports 1 parameter for *D. melanogaster* and 1 parameter for *D. ananassae/D. willistoni*. Further model testing supports 2 N:S parameters for *D. melanogaster*, 1 for divergence and 1 for polymorphism. c) Considering support for a single parameter for *D. ananassae/D. willistoni*, we found support for distinct shared parameters for divergence and polymorphism. d) Considering support for distinct parameter estimates for divergence and polymorphism shared between *D. ananassae/D. willistoni,* we find further support for distinct divergence and polymorphism for each species. Final model choice is shown with 6 parameters in red text. This justified subsequent testing in Figs. 3 and 4.

Figure 2 indicates the results from the model testing performed to capture species-level properties. Nested LRT found strong support for a model with 6 unique values of *β*, for divergence and polymorphism N:S odds for each of 3 species (Fig. 2a, final selected model parameters in red, Fig. 2b–d, structure of nested testing). Interestingly, N:S odds modifiers for divergence are significantly lower in *D. ananassae* (*β*_ana.div_ = 0.693) and *D. willistoni* (*β*_ana.div_ = 0.517) compared with *D. melanogaster* (*β*_mel.div_ = 1.22). A similar contrast with respect to these species has been independently noted with PAML (Heger and Ponting 2007; Castillo *et al*. 2011). We also found significant, but very minor, differences in the N:S odds for polymorphism, (*β*_mel.poly_ = 1.105, *β*_ana.poly_ = 1.207, *β*_wil.poly_ = 1.1313). Overall, this indicates that the genome-wide patterns of population level constraint on coding sequences are similar across species, despite differences in the N:S odds for divergence. The reason for this difference is not understood.

**Fig. 3.**
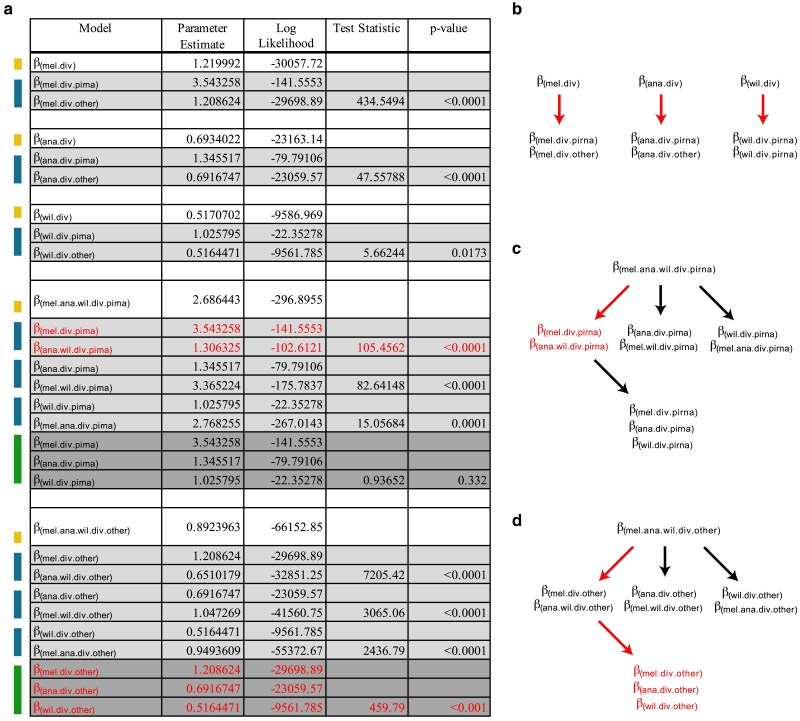
Model testing for differences in the N:S divergence odds across each of the 3 species with respect to piRNA genes and other genes. a) Colors on the left indicate nested models being tested against models indicated above. Yellow indicates a single parameter model, green indicates a 2-parameter model and blue indicates a 3-parameter model. Three-parameter models were tested against the 2-parameter model with the highest likelihood. *P*-values were determined based on the LRT statistic, using the *χ*^2^ distribution with degrees of freedom equal to the number of additional parameters. In all cases, the number of additional parameters for each test was equal to 1. Red indicates the parameters of the final model selected. Here, a distinct value for N:S odds was chosen for divergence in the piRNA machinery of *D. melanogaster* and a lower shared value was chosen for *D. willistoni* and *D. ananassae*. Unique N:S odds were selected for all other genes for each species. b–d) Structure of LRT. Red arrows indicate a nested LRT that favors an additional parameter. Red text indicates the final selected parameters for the chosen model. b) Considering support for a 6-parameter model of divergence and polymorphism for each species (Fig. 2) hypothesis testing finds support for distinct divergence parameters for the piRNA machinery of each species, compared with other genes. c) To ensure robust nested testing, we explicitly tested support for a unique divergence parameter across the piRNA machinery of each species, and find support for a unique parameter estimated for *D. melanogaster*, but a shared estimate for *D. ananassae* and *D. willistoni*. d) Further nested hypothesis testing for other genes finds support for a unique divergence parameter for each species. Final model choice is shown with 5 parameters in red text.

Next, we sought to determine how N:S divergence and polymorphism differs between components of the piRNA pathway and all other genes. Consistent with previous studies, hypothesis testing (Fig. 3) reveals significant support for a higher value of N:S divergence in the piRNA machinery of *D. melanogaster* (*β*_mel.div.piRNA_ = 3.543) compared with all other *D. melanogaster* genes (*β*_mel.div.other_ =1.209). Likewise, the selected model supports greater divergence odds modifiers for the piRNA machinery in *D. ananassae* (*β*_ana.div.piRNA_ = 1.346; *β*_ana.div.other_ = 0.692) and *D. willistoni* (*β*_wil.div.piRNA_ = 1.026; *β*_wil.div.other_ = 0.516). However, compared with other genes, there is a greater excess in the piRNA machinery of *D. melanogaster* compared with the other 2 species with greater TE load (*D. melanogaster*: *β*_mel.div.piRNA_/*β*_mel.div.other_ = 2.94; *D. ananassae*: *β*_ana.div.piRNA_/*β*_ana.div.other_ = 1.945; *D. willistoni*: *β*_wil.div.piRNA_/*β*_wil.div.other_ = 1.988). After further testing, the final selected model supported a single modifier of the N:S divergence odds in *D. melanogaster* (*β*_mel.div.piRNA_ = 3.543) and a single joint value for *D. ananassae* and *D. willistoni* (*β*_ana.wil.div.piRNA_ = 1.306). Overall, the final selected model shows a higher N:S divergence odds in the piRNA machinery of *D. melanogaster* relative to other genes in the genome, even though *D. melanogaster* has the lowest TE burden.

**Fig. 4.**
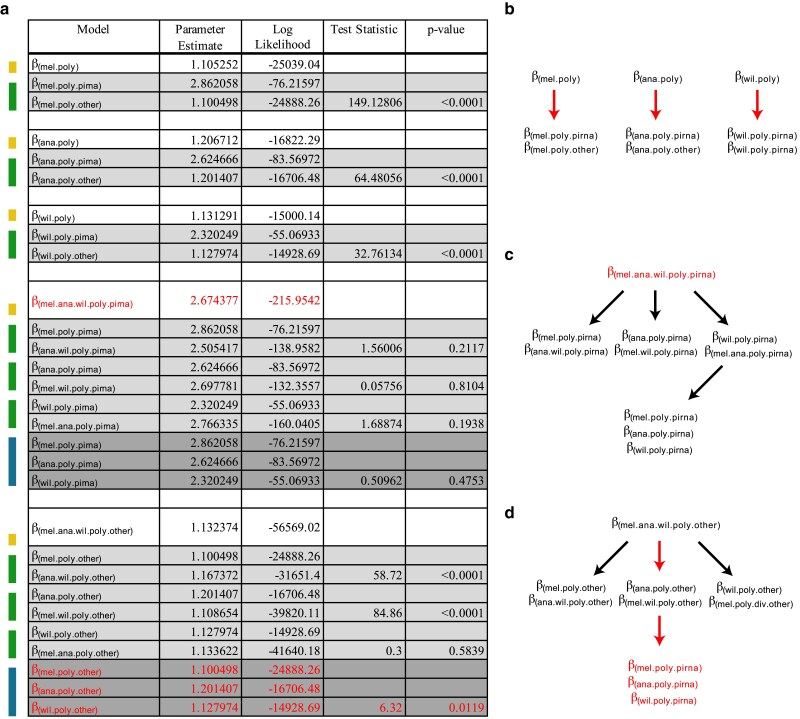
Model testing for differences in the N:S polymorphism odds across each of the 3 species with respect to piRNA genes and other genes. a) Colors on the left indicate nested models being tested against models indicated above. Yellow indicates a single parameter model, green indicates a 2-parameter model and blue indicates a 3-parameter model. Three-parameter models were tested against the 2-parameter model with the highest likelihood. *P*-values were determined based on the LRT statistic, using the *χ*^2^ distribution with degrees of freedom equal to the number of additional parameters. In all cases, the number of additional parameters for each test was equal to 1. Red indicates the parameters of the final model selected. Here, a single joint value for N:S odds was chosen for polymorphism in the piRNA machinery of all 3 species. Unique, albeit highly similar, N:S polymorphism odds were selected for all other genes for each species. b–d) Structure of LRT. Red arrows indicate a nested LRT that favors an additional parameter. Red text indicates the final selected parameters for the chosen model. b) Considering support for a 6-parameter model of divergence and polymorphism for each species (Fig. 2), hypothesis testing finds support for distinct polymorphism parameters for the piRNA machinery of each species, compared with other genes. c) To ensure robust nested testing, we explicitly tested support for a unique polymorphism parameter across the piRNA machinery of each species, but only find support for a single parameter estimated for *D. melanogaster*, *D. ananassae* and *D. willistoni.* d) Further nested hypothesis testing for other genes finds support for a unique, albeit highly similar, parameter for each species. Final model choice is shown with 4 parameters in red text.

The MK test compares divergence to polymorphism. Therefore, we separately evaluated N:S polymorphism odds across species and gene class (Fig. 4). The model supported a single modifier for the piRNA machinery of all 3 species (*β*_mel.ana.wil.poly.piRNA_ = 2.67) and distinct but similar ones for each species for the remaining genes (*β*_mel.poly.other_ = 1.100; *β*_ana.poly.other_ = 1.201; *β*_wil.poly.other_ = 1.128). In contrast to the divergence N:S odds, the N:S polymorphism odds for the piRNA machinery and other genes is similar across all 3 species.

One general concern about the approach we have taken is that we seek to obtain a single estimate for the N:S odds by aggregating data across many genes, either the piRNA machinery or all other genes in the genome. This can be problematic for several reasons. First, if there is underlying variation in the N:S odds across genes, then genes with more variant counts will influence the estimate of N:S more than genes with few variant counts. For example, consider this possibility for divergence. Suppose 1 piRNA gene had 100 divergent variants, 75 of which were nonsynonymous and 25 were synonymous. If 30 remaining piRNA genes each had 2 divergent variants (1 nonsynonymous and 1 synonymous), the N:S estimate would be overwhelmingly driven by the 1 single gene with 100 divergent variants. One could very reasonably argue that this is problematic. However, it may be noted that if one is less interested in estimating an average of N:S odds across each individual gene, and more interested in measuring the total proportion of nonsynonymous divergence, relative to synonymous divergence, across an entire pathway, then singular codons across the pathway, however distributed among different genes, may be more relevant. A related concern is Simpson's paradox. By summing contingency tables across genes (this approach bins counts across genes to estimate N:S odds), even if individually each table has the same odds-ratio, one can obtain odds-ratios that are different (Shapiro *et al*. 2007; Stoletzki and Eyre-Walker 2011). To mitigate against these problems, we took several approaches. First, we performed gene-wise bootstrapping to ensure results were not driven by a few genes with many counts and sought to determine how the N:S divergence to N:S polymorphism odds-ratio varies as a function of gene class. In particular, for each species, we sought to estimate the following parameter *χ*:


$$\chi \;=\;\frac{\frac{\mathrm{Odds}.\mathrm{Divergence}.\mathrm{piRNA}}{\mathrm{Odds}.\mathrm{Polymorphism}.\mathrm{piRNA}}}{\frac{\mathrm{Odds}.\mathrm{Divergence}.\mathrm{Other}}{\mathrm{Odds}.\mathrm{Polymorphism}.\mathrm{Other}}}$$

**Fig. 5.**
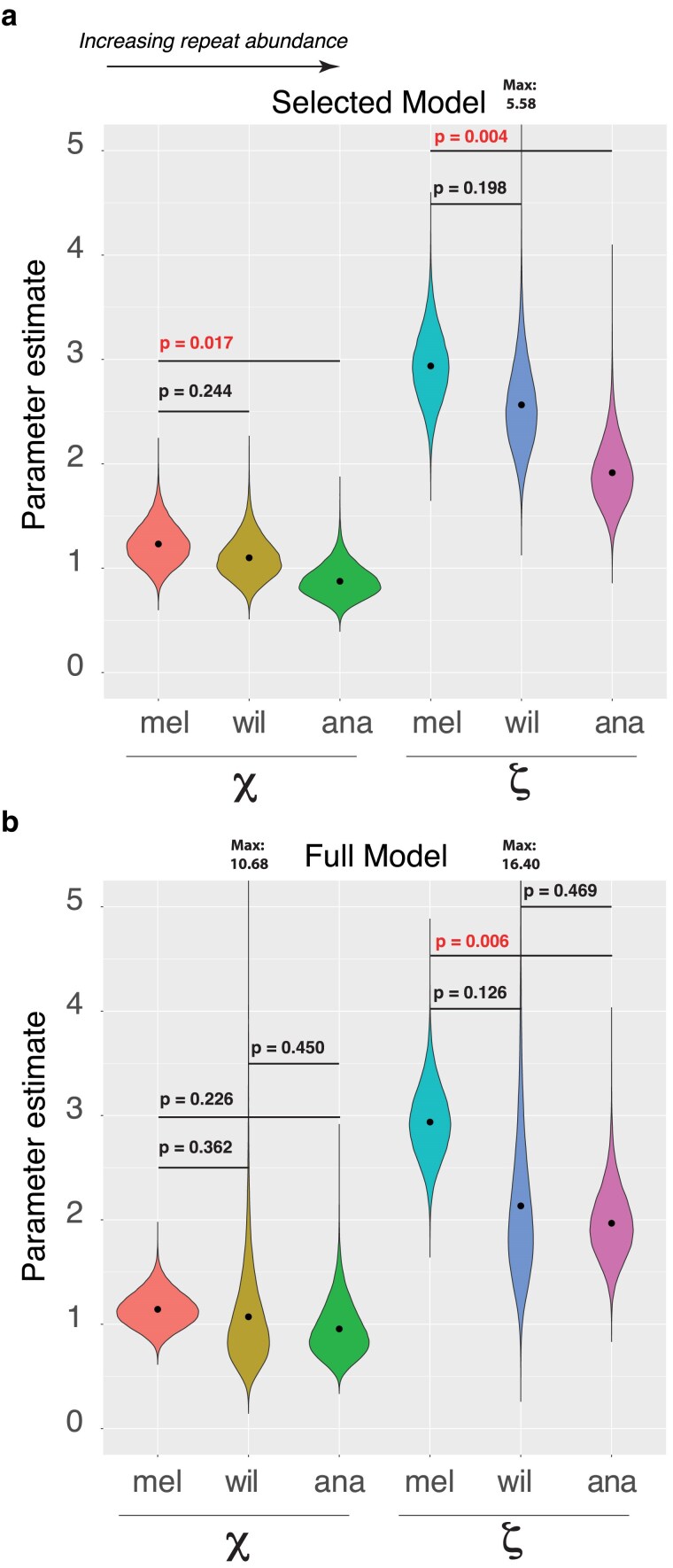
Bootstrap distributions and significance testing of summary statistics. a) Bootstrap distributions of summary statistics under the selected model, mean indicated by black point. *P*-values were determined by determining the number of times a given permutation of piRNA genes and other genes provided a summary statistic difference between 2 species in the opposing direction to what was observed in estimates provided by the selected model. b) Bootstrap distributions of summary statistics under the full 12-parameter model, mean indicated by black point, *P*-values were determined by determining the number of times a given permutation of piRNA genes and other genes provided a summary statistic difference between 2 species in the opposing direction to what was observed estimates provided by the full model.

We obtained ML estimates of *χ* for each permutation (each permutation was obtained by sampling, with replacement, the 31 piRNA genes and, separately, the other genes) under the selected model (Fig. 5a). The estimate of *χ* is lowest in *D. ananassae,* intermediate in *D. willistoni* and highest in *D. melanogaster*. The bootstrap estimate for *χ* obtained from the selected model for *D. ananassae* is significantly lower than that of *D. melanogaster* (*χ*_mel_ = 1.232; *χ*_ana_ = 0.874 ; *P* = 0.01658). Even though the estimate of *χ* for *D. willistoni* was also lower than that of *D. melanogaster*, the difference was not significant (*χ*_wil_ = 1.099; *P* = 0.2437). Under the selected model, these results reject a higher value of *χ* in *D. ananassae* vs *D. melanogaster*.

Excluding polymorphism, we also considered estimates of the odds-ratio for branch-specific N:S divergence in the piRNA machinery relative to other genes. We designate this odds-ratio *ζ*:


$$\zeta \;=\;\frac{\mathrm{Odds}.\mathrm{Divergence}.\mathrm{piRNA}}{\mathrm{Odds}.\mathrm{Divergence}.\mathrm{Other}}$$

Figure 5a shows how this estimate varies across the 3 species. As seen for *χ*, estimates of *ζ* in *D. ananassae* are significantly lower than that of *D. melanogaster* (*ζ*_mel_ = 2.937; *ζ*_ana_ = 1.915; *P* = 0.00374). Again, estimates for *D. willistoni* are intermediate, though not significantly lower than that of *D. melanogaster* (*ζ*_wil_ = 2.565; *P* = 0.1979).

Due to the limitations of the selected model, with a shared joint estimate of polymorphism odds between *D. ananassae* and *D. willistoni*, we also evaluated estimates of *χ* and *ζ* under a full model where estimates were obtained only from data from individual species. As expected (Fig. 5b), variances in the estimates are increased due to reduced precision for estimates of polymorphism odds for the piRNA machinery in *D. ananassae* and *D. willistoni*. Thus, for the full model, no contrast in estimates of *χ* (*χ*_mel_ = 1.143; *χ*_anal_ = 0.955; *χ*_wil_ = 1.072) across species is significant (*χ*_mel_ vs *χ*_ana_, *P* = 0.2257; *χ*_mel_ vs *χ*_wil_, *P* = 0.363; *χ*_ana_ vs *χ*_wil_, *P* = 0.4506). However, when only *ζ* is evaluated, the estimate for *D. ananassae* (*ζ*_ana_ = 1.968) is significantly less than that of *D. melanogaster* (*ζ*_mel_ = 2.937; *P* = 0.006). Other contrasts were not significant (*ζ*_mel_ = 2.937 vs *ζ*_wil_ = 2.134, *P* = 0.126; *ζ*_anal_ = 1.968 vs *ζ*_wil_ = 2.134, *P* = 0.468). Thus, even though the full model was not selected by LRTs, the estimate of *ζ* for *D. ananassae* is significantly lower than that of *D. melanogaster*. Thus, we can conclude that for *D. ananassae* and *D. melanogaster*, increased TE content is associated with a lower rate of nonsynonymous divergence in the piRNA machinery compared with other genes.

This bootstrapping approach does not fully mitigate against Simpson's paradox if, for example, a modest number of genes with high variant counts, as a group, are skewed toward a higher N:S ratio. Again, this is a concern if one is seeking to obtain an average of the N:S odds across a number of genes and is not directly interested in simply the bulk N:S odds across an entire pathway, independent of how this divergence is distributed across the genes in the pathway. One way to mitigate against bias in the estimate of the average value of the neutrality index (*NI*) across a group of genes has been proposed, so we examined this as well (Stoletzki and Eyre-Walker 2011). The *NI* is defined as (*P_n_*/*P_s_*)/(*D_n_*/*D_s_*) where *P* and *D* indicate polymorphism and divergence, respectively, for nonsynonymous (*n*) and synonymous (*s*) variants (Rand and Kann 1996). A version of the *NI* designated *NI_TG_* (Stoletzki and Eyre-Walker 2011) provides a weighted average of the *NI* across multiple genes and has reduced bias in estimating the average odds-ratio. In our case, we are interested in the inverse of *NI* (*D_n_*/*D_s_*)/(*P_n_*/*P_s_*), so we determined 1/*NI_TG_* using this approach. There is low divergence for *D. willistoni*, leading to an excess of zero values, so we focused solely on 1/*NI_TG_* for *D. melanogaster* and *D. ananassae.* The estimate of 1/*NI_TG_* for the piRNA machinery of *D. melanogaster* is 1.238 and, for *D. ananassae*, is 0.856. Since values of 1/*NI_TG_* >1 indicate an excess of nonsynonymous divergence, and values <1 indicate a depletion, this supports the conclusion that despite lower repeat abundance, *D. melanogaster* has a stronger signature of positive selection in the piRNA pathway. However, consistent with our earlier results, for all other genes in *D. melanogaster,* we also estimate a value of 1/*NI_TG_* (0.88) that is higher than that observed for *D. ananassae* (0.566). If we normalize to these genome wide values, (1/*NI*_TG.piRNA_)/(1/*NI*_TG.other_) is greater in *D. ananassae* (1.513) compared with *D. melanogaster* (1.401). Nonetheless, while the *D. ananassae* piRNA machinery shows a greater amount of nonsynonymous divergence compared with background, a value of 1/*NI*_TG_ for the piRNA machinery of *D. melanogaster* that is about 45% greater than that in *D. ananassae* is not consistent with the hypothesis that higher repeat content in *D. ananassae* drives a higher rate of positive selection compared with *D. melanogaster*.

**Fig. 6.**
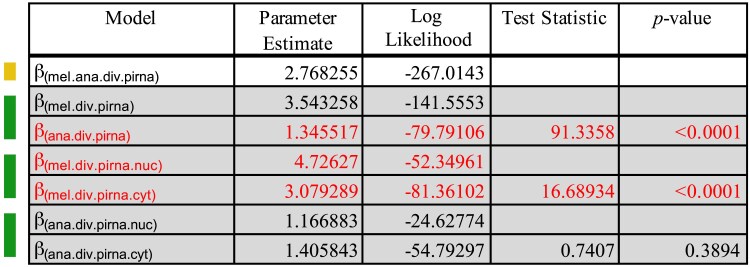
Model testing for differences in the N:S divergence odds between cytoplasmic and nuclear components of the piRNA machinery in *D. melanogaster* and *D. ananassae*. Colors on the left indicate nested models being tested against models indicated above. Yellow indicates a single parameter model and green indicates a 2-parameter model. Red indicates the parameters of the final model selected. Here, a single joint value for N:S odds was chosen for both nuclear and cytoplasmic piRNA machinery divergence in *D. ananassae*. However, unique values were selected for the nuclear and cytoplasmic components of the piRNA machinery in *D. melanogaster*. After normalizing to genome-wide divergence estimates, it is clear that the difference in estimates of *ζ* between *D. melanogaster* and *D. ananassae* is primarily driven by nuclear components of the piRNA machinery.

Finally, we sought to determine if evolution might be distinct between nuclear and cytoplasmic components of the piRNA machinery (see Materials and methods for designations). Focusing only on divergence and again excluding *D. willistoni* due to low divergence, we performed LRTs to identify the preferred model, considering modifier parameters for species *and* cellular compartments (Fig. 6). The selected model favored a single parameter value for *D. ananassae* piRNA machinery (1.3455) and distinct values for the *D. melanogaster* piRNA machinery in the cytoplasm (3.0792) and the nucleus (4.7276). By normalizing these values against the background estimates, we see that the nuclear piRNA machinery of *D. melanogaster* contributes most to the differences between *D. melanogaster* and *D. ananassae ζ* for the piRNA machinery (*ζ*_ana.piRNA_ = 1.95, *ζ*_mel.cyt_ = 2.55, *ζ*_mel.nuc_ = 3.91).

## Discussion

Multiple studies have shown that the piRNA machinery of *Drosophila* has a strong signature of positive selection, but the cause of this pattern is unknown. Here, we develop a hypothesis testing approach to test the hypothesis that the piRNA machinery will show a greater signature of positive selection in species with greater TE content. This approach is a bit different than other extensions of the MK test, which seek to measure key parameters of natural selection. For example, a population genetic framework known as the Poisson random field can use a 2×2 table of divergence and polymorphism to obtain an estimate of the selection parameter *γ*, which is the selection coefficient multiplied by the effective population size (Sawyer and Hartl 1992; Bustamante *et al*. 2002; Sawyer *et al*. 2007). 2×2 tables of divergence and polymorphism have also been used to estimate the proportion of amino acid substitutions driven by positive selection (Smith and Eyre-Walker 2002) and a metric designated the direction of selection (Stoletzki and Eyre-Walker 2011). The approach here is somewhat different because it does not estimate population genetic parameters. Rather, it takes a simpler approach to nested hypothesis testing for differences in the N:S odds. In this way, it is generalizable to different levels of nested hypothesis testing, as demonstrated by how we tested whether different components of the piRNA machinery (cytoplasmic or nuclear) contribute to the estimate of the N:S odds. However, one must be cautious in the interpretation. As discussed, a single estimate of the N:S odds for a set of genes will be most strongly influenced by genes with greater counts. A solution to this is to use an approach such as previously proposed (Stoletzki and Eyre-Walker 2011) to obtain an estimate of the average OR across multiple genes, but this approach lacks a clear nested likelihood-based hypothesis testing approach. Therefore, it is important to be aware that the estimate from the approach introduced here may not be an estimate from a set of genes with a uniform N:S parameter. Rather, the approach characterizes the bulk divergence and polymorphism across a set of genes as if it was concatenated into a single gene. This concatenated set approach is also employed for the asymptotic MK test (Messer and Petrov 2013; Haller and Messer 2017), which combines count data across many genes. In light of these approaches, it is interesting to consider how combining results from 2 different functional domains of a single gene in a MK test may also represent something similar to summing 2 different contingency tables. For a worthwhile discussion of comparing odds across different data partitions, see: https://dynomight.net/simpsons-paradox/.

From this study, we make 2 primary conclusions. First, the piRNA machinery in *D. melanogaster* shows a significant excess of N:S divergence compared with *D. ananassae* and *D. willistoni.* In addition, the *χ* parameter that compares the evolution of the piRNA machinery to all other genes, normalized by polymorphism, is greatest in *D. melanogaster* and lowest in *D. ananassae,* a species with higher TE content. Ignoring underlying polymorphism, both the selected model and the full model have estimates of *ζ* that are higher than 1 for each of these 3 species (Fig. 5). These results are consistent with a high rate of adaptation in the piRNA machinery of all species, and further, they provide strong support for a difference in the strength of selection acting on the piRNA machinery of *D. melanogaster* compared with *D. ananassae* and *D. willistoni*. These results are also similar to those previously noted, with a lower estimate of piRNA machinery *dN*/*dS* in species with higher TE content (Castillo *et al*. 2011).

We consider several different models that may explain rapid adaptive evolution in the piRNA machinery, and whether these results reject any particular model or, perhaps, simply provide support for 1 model over the others. The first of these is the TE co-evolutionary arms race model. This model proposes that adaptation in the piRNA machinery is driven by rounds of antagonistic co-evolution between the piRNA machinery and 1 or multiple TE families. A second related model, though not co-evolutionary, is that the piRNA machinery must constantly adapt to novel strategies employed by newly invading TEs. A third model is the genomic autoimmunity hypothesis. It proposes that, as TE activity ebbs and flows, selection fluctuates in the piRNA machinery between sensitivity to new TEs and specificity that prevents off-target gene silencing. Finally, a fourth model proposes rapid adaptation in the piRNA machinery unrelated to TEs but is instead due to other functions of the piRNA machinery, such as regulating meiotic drive.

These results are not consistent with a simplistic model of TE-piRNA machinery co-evolution whereby increased total TE abundance (distinct from TE activity and TE diversity) is the driver of positive selection. However, results are consistent with other more complex versions of this model. In particular, there may be ongoing antagonistic co-evolution between, say, 2 TE families and the piRNA machinery of *D. melanogaster*, but, for whatever reason, such co-evolution may only occur with 1 TE family each in *D. ananassae* and *D. willistoni*. In this case, adaptation in the piRNA machinery would be driven by antagonistic co-evolution, but the strength of the effect on the piRNA machinery would be greatest in *D. melanogaster*, which also happens to have lower TE abundance.

These results are also not consistent with a simplistic genomic autoimmunity hypothesis. A naïve version of this hypothesis would suggest that adaptation in the piRNA machinery would occur as TE activity both increases and decreases and is thus uniform across species. A species with a high TE burden might be experiencing a phase of decreasing activity, and a species with a low TE burden might be experiencing a phase of increasing TE activity. Under a simple model, one might predict that this mode of fluctuating selection might be ongoing and thus *independent* of total current genomic abundance. However, the results here, combined with a previous study (Castillo *et al*. 2011), indicate that adaptation in the piRNA machinery may not be independent of TE burden. In fact, higher TE burden seems to slow down adaptation. Nonetheless, as with the co-evolution model, a more realistic version of the genomic autoimmunity hypothesis might still be consistent with these results. For example, *D. melanogaster* may be experiencing a recent increase in TE activity, but *D. ananassae* and *D. willistoni* may be at equilibrium. In this case, higher levels of adaptation in the *D. melanogaster* piRNA machinery may be driven by a newly increased premium on sensitivity in the piRNA machinery for detecting newly invading TEs.

We also consider 2 other models to explain the high rate of adaptation in the piRNA machinery. The first is that while co-evolution may not be occurring, the piRNA machinery still experiences antagonism on the part of a newly invading TE family and natural selection on the piRNA machinery acts to evade this antagonism. This is similar to the co-evolutionary hypothesis but does not require corresponding adaptation in the TE lineage. The second model is that adaptation is driven in the piRNA machinery not by TEs, but by bouts of meiotic drive that come under the control of the piRNA machinery. The *Stellate* system of “ancient meiotic drive” may represent such a system. If similar systems are absent in *D. ananassae* and *D. willistoni*, this may explain increased adaptation in the piRNA machinery of *D. melanogaster*. However, the results here do not directly speak to either of these models.

Overall, the results of this study highlight a challenge in using evolutionary approaches, rather than mechanistic ones, to distinguish between models that explain the high rate of adaptation in the piRNA machinery of *D. melanogaster*. This may be because a singular global proxy for “TE challenge” might be inappropriate. Perhaps instead of multiple TE families jointly contributing to patterns of molecular evolution, a single active family does. More broadly, an implicit assumption for the hypothesis tests here was that the standing estimates of TE load and adaptation are the results of a long-term equilibrium processes. The simplistic model of antagonistic co-evolution that proposed a higher rate of adaptation in *D. ananassae* and *D. willistoni* assumes that all 3 species are in equilibrium, with those carrying a higher TE load experiencing an increased equilibrium rate of adaptation. The simplistic model of the genomic autoimmunity hypothesis relied on the assumption that, independent of current TE load, all species will be experiencing similar levels of fluctuating selection between sensitivity and specificity, resulting in some stable equilibrium of adaptation that is uniform across species.

Importantly, the assumption of equilibrium in evaluating these models may be problematic. *D. melanogaster* has experienced recent range expansion out of Africa and results here and in previous studies (Heger and Ponting 2007; Castillo *et al*. 2011) indicate that the N:S odds for divergence is greater in *D. melanogaster* than *D. ananassae* and *D. willistoni*. Perhaps this is due to adaptation during range expansion. Additionally, while little is known about the history of TE dynamics in *D. willistoni* and *D. ananassae*, non-equilibrium TE dynamics are clearly apparent in *D. melanogaster*. This has been known since the discovery of the *P*-element invasion in *D. melanogaster* that occurred during the 1950s (Anxolabehere *et al*. 1988). Multiple syndromes of hybrid dysgenesis reveal a non-equilibrium pattern of invasion, suppression, and re-invasion (Blumenstiel 2019). Studies of old strains and museum samples (Schwarz *et al*. 2021; Scarpa *et al*. 2024) have also shown that *Tirant*, *Blood*, *Opus,* and *412* elements have also invaded *D. melanogaster* within the past 200 years. Perhaps range expansion in *D. melanogaster*, coupled with new TE invasions, has led to shifts of selection both genome-wide and *especially* on the piRNA machinery. Despite having a high TE burden*, D. ananassae* and *D. willistoni* may not have been exposed to similar recent shifts. Thus, *D. melanogaster* might be an outlier here.

Whether this signature of increased adaptation in the piRNA machinery of *D. melanogaster* is driven by a co-evolutionary arms race, recent TE invasion, genomic autoimmunity or TE-independent mechanisms awaits joint investigation of the timing of adaptation and the timing of TE proliferation. This may be achieved by comparing patterns of TE invasion with the history of adaptation in the piRNA machinery. Recent advances in estimating the ancestral recombination graph (Deng *et al*. 2024; Lewanski *et al*. 2024; Nielsen *et al*. 2025) may provide important insight into how and when new alleles in the piRNA machinery were selected in the face of TE invasions. A molecular analysis of the function of these selected alleles is also likely to provide further insight.

## Supplementary Material

jkaf017_Supplementary_Data

## Data Availability

Sequence data is available under BioProject IS PRJNA1210691. Alignments are provided at GSA FigShare at https://doi.org/10.25387/g3.28266422. Variant tables and R program are provided in Supplementary materials. Supplemental material available at G3 online.
